# Trends and Projected Burden of HIV/AIDS in Kazakhstan, 2010–2030: A Comparative Analysis Using GBD 2023 Estimates

**DOI:** 10.3390/tropicalmed11070171

**Published:** 2026-06-24

**Authors:** Indira Karibayeva, Gulzar Shah, Nikolay Lunchenkov, Roza Kuanyshbekova, Kuanysh Shonbay, Botagoz Turdaliyeva

**Affiliations:** 1 Department of Health Policy and Community Health, Jiann-Ping Hsu College of Public Health, Georgia Southern University, Statesboro, GA 30460, USA; ik01379@georgiasouthern.edu; 2 Department of Research Management, JSC Research Institute of Cardiology and Internal Diseases, Almaty 050012, Kazakhstan; 3 TUM School of Social Sciences and Technology, Technical University of Munich, 80333 Munich, Germany; nikolay.lunchenkov@tum.de; 4 Health and Capacity Building Department, Eurasian Coalition on Health, Rights, Gender and Sexual Diversity, 10151 Tallinn, Estonia; 5 Kazakh Scientific Center of Dermatology and Infectious Diseases, Almaty 050008, Kazakhstan; r.kuanyshbekova@kncdiz.kz; 6 Higher School of Psychology, Turan University, Almaty 050013, Kazakhstan; shonbaykuanysh@gmail.com; 7 Department of Nursing, Asfendiyarov Kazakh National Medical University, Almaty 050012, Kazakhstan

**Keywords:** HIV/AIDS, Kazakhstan, Global Burden of Disease, epidemiology, incidence, prevalence, DALYs, HIV prevention, PrEP, Central Asia

## Abstract

Background: HIV/AIDS remains a major global public health challenge, with persistent regional disparities in burden and progress toward the UNAIDS 95–95–95 targets. This study assessed temporal trends in the HIV/AIDS burden in Kazakhstan, compared them with Central Asia and global patterns, and projected trends through 2030. Methods: We conducted a population-level analysis using Global Burden of Disease 2023 data, examining age-standardized rates (per 100,000) of incidence, prevalence, mortality, disability-adjusted life years (DALYs), years of life lost (YLLs), and years lived with disability (YLDs) from 2010 to 2023. Trends were quantified using percent change and average annual percentage change, with projections based on log-linear models. Results: Between 2010 and 2023, prevalence in Kazakhstan increased by 332.1% and incidence by 111.0%, contrasting with the decline in global incidence (−24.7%). Mortality decreased (−32.7%), along with DALYs (−28.8%) and YLLs (−37.1%), while YLDs increased by 135.5%, indicating a shift toward a chronic disease burden. In 2023, Kazakhstan had a lower overall burden than global estimates but showed steeper increases in incidence and prevalence. Age-specific analyses indicated the largest increases among adults aged 30–69 years. Under current trend assumptions, projections suggest continued growth in prevalence and incidence, with modest mortality declines through 2030, though these trajectories do not account for future changes in prevention coverage, treatment access, or policy. Conclusions: Kazakhstan is undergoing a transition toward a chronic HIV epidemic, underscoring the need to strengthen prevention, expand PrEP and testing coverage, and address structural barriers to achieve epidemic control.

## 1. Introduction

Human immunodeficiency virus (HIV) infection remains a major global public health challenge despite substantial progress in prevention, diagnosis, and treatment. In 2024, an estimated 40.8 million people worldwide were living with HIV, with 1.3 million new infections and 630,000 AIDS-related deaths reported that year [1]. While global age-standardized mortality and disability-adjusted life years (DALYs) attributable to HIV have declined due to the scale-up of antiretroviral therapy (ART), the burden has shifted toward chronic disease management, reflected in rising prevalence driven by improved survival. By 2024, 31.6 million people were accessing ART, yet regional disparities remain stark [1]. The Joint United Nations Programme on HIV/AIDS (UNAIDS) set the 95–95–95 targets for 2030, but as of 2023, only 86% of people living with HIV globally knew their status, underscoring gaps in diagnosis and linkage to care [2].

A critical but often underemphasized challenge in HIV epidemiology—particularly in Eastern Europe and Central Asia (EECA)—is the high prevalence of late diagnosis [3,4,5]. A considerable share of newly recorded cases reflects delayed detection of infections acquired years earlier, rather than true recent transmission. Consequently, estimates of HIV incidence derived from surveillance and modeling frameworks may partially capture this diagnostic delay, complicating the interpretation of temporal trends in incidence and obscuring the distinction between ongoing transmission and improved case detection.

EECA is one of the few regions where HIV incidence continues to rise. In 2023, 140,000 new HIV infections occurred in EECA, representing a 20% increase since 2010, with Kazakhstan, Russia, Ukraine, and Uzbekistan accounting for over 90% of new cases [6]. Unlike other regions where incidence and mortality are declining, EECA remains off track to meet epidemic control targets, with 94% of new infections concentrated among key populations and their sexual partners.

Kazakhstan, the largest country in Central Asia, has undergone notable shifts in HIV epidemiology over the past two decades. Epidemiological evidence indicates a steady increase in HIV prevalence, particularly among key populations, including people who use drugs, sex workers, men who have sex with men (MSM), and migrant populations [7,8,9]. Population mobility, urbanization, and persistent stigma affecting key populations continue to sustain transmission, while ongoing health system reforms and broader access to ART create opportunities to improve HIV prevention and care [3,10,11]. Existing studies in Kazakhstan have largely focused on subpopulations or localized settings, with limited comprehensive assessments of the long-term, population-level burden.

Despite Kazakhstan’s rising HIV prevalence, no study has comprehensively quantified temporal trends in incidence, prevalence, mortality, and DALYs using standardized metrics, nor has any study systematically compared Kazakhstan’s burden with Central Asia and global estimates.

Given these gaps, a comprehensive evaluation of the HIV/AIDS burden in Kazakhstan using standardized, comparable metrics is warranted. Therefore, this study aimed to (1) quantify temporal trends in age-standardized HIV/AIDS incidence, prevalence, mortality, and composite burden measures from 2010 to 2023 across Kazakhstan, Central Asia, and global estimates; (2) assess sex-specific and age-specific variations in burden; and (3) project future trajectories of the HIV/AIDS burden in Kazakhstan through 2030.

## 2. Materials and Methods

### 2.1. Data Source and Study Framework

This study was designed as a population-level epidemiological analysis of HIV/AIDS burden using data from the Global Burden of Disease (GBD) 2023 study, produced by the Institute for Health Metrics and Evaluation. The GBD framework synthesizes data from multiple sources, including vital registration systems, surveillance data, and epidemiological studies, to generate standardized and comparable estimates of disease burden across countries and over time. A comprehensive description of the GBD data collection and estimation methodology has been published previously [12,13,14].

Data were extracted from the GBD Results Tool (https://vizhub.healthdata.org/gbd-results/) accessed on 28 February 2026 using the following filters: cause = HIV/AIDS (cause ID 298); measures = incidence, prevalence, deaths, DALYs, YLLs, and YLDs; metric = rate (per 100,000 population); locations = Kazakhstan, Central Asia (GBD super-region), and Global; years = 2010–2023; age groups = all ages (age-standardized) and predefined five-year GBD age bands from 0 to 14 to 70+ years; sex = male, female, and both sexes combined. Age-standardized rates were computed by the GBD using the GBD standard population (a theoretical population based on the average age structure across all countries included in the study). Both age-standardized rates and age-specific rates were analyzed; counts were not the primary outcome of this analysis. All GBD estimates include 95% uncertainty intervals (UIs), reflecting model-based statistical uncertainty in data inputs and estimation. These UIs were reported descriptively alongside point estimates but were not propagated into the APC log-linear regression models or projection analyses, as the GBD does not provide draw-level data in the public results tool at the resolution required for full uncertainty propagation.

We extracted HIV/AIDS-specific estimates for Kazakhstan, Central Asia, and the global population for the period 2010–2023. The primary outcomes included age-standardized rates per 100,000 population of incidence, prevalence, mortality, disability-adjusted life years (DALYs), years of life lost (YLLs), and years lived with disability (YLDs). Age-standardized rates were derived using the GBD standard population. All estimates were analyzed separately for males, females, and both sexes combined.

Age-specific analyses were conducted using predefined GBD age groups ranging from 0 to 14 years to 70+ years in 5-year intervals. All GBD estimates were reported with corresponding 95% uncertainty intervals (UIs), reflecting uncertainty associated with data inputs and model estimation.

### 2.2. Analytical Strategy and Modeling Procedures

All statistical analyses were conducted using R statistical software, version 4.5.1 (R Foundation for Statistical Computing, Vienna, Austria). Descriptive analyses were performed to summarize HIV/AIDS burden across locations, sex groups, age groups, and calendar years. Outcomes included age-standardized and age-specific rates per 100,000 population for incidence, prevalence, mortality, disability-adjusted life years (DALYs), years of life lost (YLLs), and years lived with disability (YLDs). Temporal changes between 2010 and 2023 were quantified using both absolute change and percent change to avoid overinterpretation of large relative changes arising from low baseline rates. To assess long-term trends, we estimated the annual percent change (APC) using log-linear regression models, in which the natural logarithm of each rate was regressed on calendar year. APC was calculated as $(e^{\beta }-1)\times 100$, where β represents the yearly change in the log-transformed rate. Statistical inference was based on 95% confidence intervals derived from the regression models. Comparative analyses were conducted to evaluate patterns across Kazakhstan, Central Asia, and global estimates. Sex-stratified analyses were used to assess differences between males and females, and age-specific analyses were performed to examine heterogeneity across the life course. Results were visualized using line plots, bar charts, and heatmaps, with uncertainty intervals incorporated where appropriate. Because constant-rate assumptions are unlikely to hold in HIV epidemiology, given changes in testing coverage, COVID-19 disruptions, ART scale-up, and PrEP introduction, projection analyses were treated as exploratory. Primary projections of age-standardized HIV/AIDS rates in Kazakhstan through 2030 were generated using log-linear extrapolation from observed 2010–2023 data and interpreted as “if-current-trends-continue” scenarios rather than forecasts of expected epidemiological reality. Prediction intervals were presented to reflect uncertainty around extrapolated values. Sensitivity analyses were conducted by refitting projection models using the more recent 2015–2023 period to assess whether projected trajectories differed when older data were excluded. This sensitivity analysis was intended to partially account for more recent changes in the HIV prevention and treatment environment, including expanded ART access, evolving testing practices, COVID-19-related disruptions, and the early post-PrEP implementation period. Because only two post-PrEP years were available in the observed GBD series, the models could not formally estimate the independent population-level effect of PrEP. Therefore, all 2030 projections were interpreted cautiously and used only to illustrate potential future trajectories under alternative trend assumptions.

## 3. Results

### 3.1. HIV/AIDS Burden in Kazakhstan and Comparator Regions in 2023

**Figure 1:**
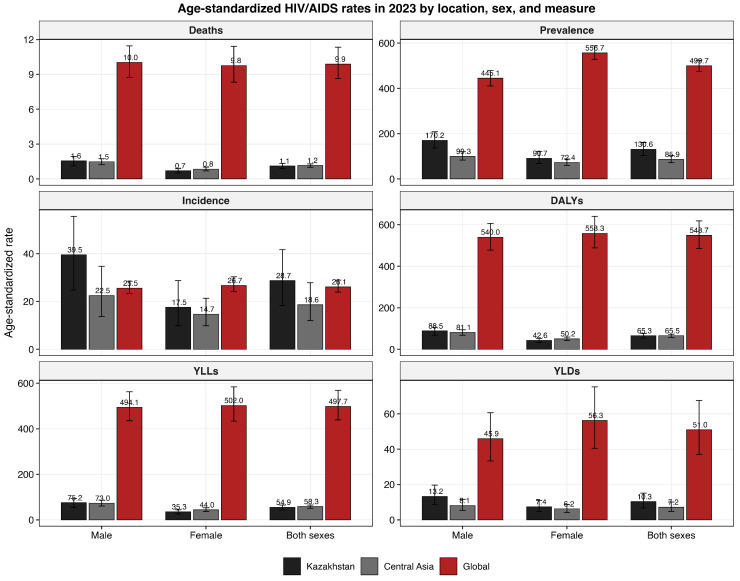
Age-standardized HIV/AIDS rates in 2023 by location, sex, and measure. Abbreviations: UI, uncertainty interval; DALYs, disability-adjusted life years; YLLs, years of life lost; YLDs, years lived with disability.

In 2023, substantial geographic and sex-based disparities in HIV/AIDS burden were observed across Kazakhstan, Central Asia, and globally (Figure 1). Globally, age-standardized rates were markedly higher than those in Kazakhstan and Central Asia across all measures. For example, among both sexes, the global age-standardized DALY rate reached 548.7 (95% UI: 485.1–618.4), compared with 65.3 (95% UI: 53.9–75.5) in Kazakhstan and 65.5 (95% UI: 57.1–72.5) in Central Asia. Similarly, global prevalence remained substantially elevated at 499.7 (95% UI: 474.8–524.3), compared with 130.6 (95% UI: 103.8–160.9) in Kazakhstan and 85.9 (95% UI: 71.4–103.4) in Central Asia. Sex-specific differences were also evident. Among males in Kazakhstan, the 2023 prevalence rate reached 170.2 (95% UI: 136.8–208.3), compared with 90.7 (95% UI: 67.9–121.5) among females, indicating a substantially higher burden in men. Comparable sex disparities were observed across Central Asia and globally.

### 3.2. Temporal Trends from 2010 to 2023

Between 2010 and 2023, the HIV/AIDS burden in Kazakhstan demonstrated substantial changes across all major indicators (Table 1). Age-standardized prevalence increased markedly, rising from 30.2 (95% UI: 22.3–39.4) to 130.6 (103.8–160.9) per 100,000 among both sexes, corresponding to an absolute increase of approximately 100 cases per 100,000 population. Similarly, incidence increased from 13.6 (10.9–16.6) to 28.7 (18.3–41.7) per 100,000, representing an absolute increase of approximately 15 cases per 100,000. These increases occurred alongside improvements in mortality indicators, with age-standardized death rates declining from 1.7 (1.5–1.9) to 1.1 (0.9–1.3) per 100,000, and reductions observed in DALYs and YLLs. In contrast, global trends were characterized by declining incidence and more pronounced reductions in mortality, while Central Asia showed intermediate patterns. Sex-specific analyses revealed important differences in both magnitude and interpretation. Although prevalence among females increased by 412.8%, this change should be interpreted in the context of a low baseline rate of 17.7 per 100,000 in 2010, rising to 90.7 per 100,000 in 2023 (absolute increase ≈73 per 100,000). In comparison, male prevalence increased from 43.4 to 170.2 per 100,000 (absolute increase ≈127 per 100,000), indicating a larger absolute burden among males despite smaller relative growth. Similarly, female incidence increased from 8.7 to 17.6 per 100,000, while male incidence rose from 18.6 to 39.5 per 100,000. These findings highlight that large percentage increases among females are partly driven by low baseline values and should not be interpreted as exceeding the absolute burden observed in males.

**Figure 2:**
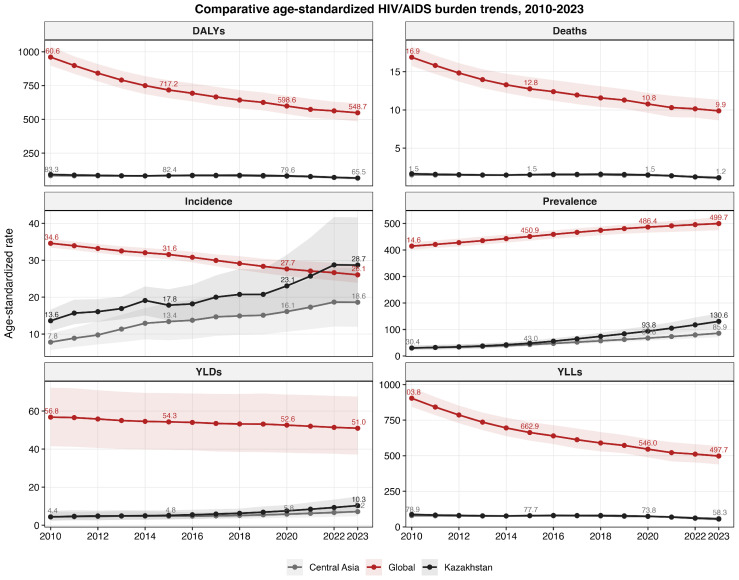
Comparative age-standardized HIV/AIDS burden trends, 2010–2023. Abbreviations: UI, uncertainty interval; DALYs, disability-adjusted life years; YLLs, years of life lost; YLDs, years lived with disability.

Temporal trend analysis confirmed these patterns (Table 2; Figure 2). In Kazakhstan, prevalence increased rapidly, with an APC of 12.7% (95% CI: 12.1 to 13.4) among both sexes, compared with 8.8% in Central Asia and 1.5% globally. Among females, prevalence increased even more sharply in Kazakhstan (APC: 14.4%, 95% CI: 13.5 to 15.3). Incidence trends also showed sustained increases in Kazakhstan (APC: 5.4%, 95% CI: 4.5 to 6.2) and Central Asia (6.4%), whereas global incidence declined significantly (APC: −2.2%). Conversely, mortality and fatal burden indicators declined. In Kazakhstan, the age-standardized death rate decreased annually by 1.8% (95% CI: −2.9 to −0.7), while DALYs declined by 1.7% and YLLs by 2.4% per year. These reductions were less pronounced than those observed globally, where DALYs declined by 4.1% annually and YLLs by 4.4%. Notably, YLDs increased significantly in Kazakhstan (APC: 6.5%, 95% CI: 5.4 to 7.6), indicating a growing contribution of non-fatal disease burden over time.

These trends are visually illustrated in Figure 2, where Kazakhstan shows increasing prevalence and incidence alongside declining mortality and DALYs, contrasting with global patterns of declining incidence and more rapid reductions in the fatal burden.

### 3.3. Age-Specific Patterns

**Figure 3:**
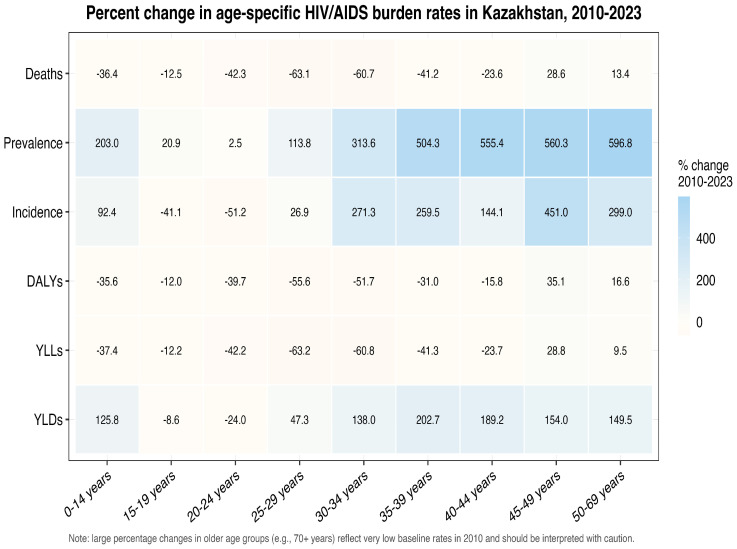
Percent change in age-specific HIV/AIDS rates in Kazakhstan, 2010–2023 (both sexes). Note: Extreme percentage changes in older age groups reflect very low baseline values and should be interpreted with caution.

Age-specific analyses revealed substantial heterogeneity across age groups and regions (Figure 3). In Kazakhstan, the largest relative increases in prevalence were observed in middle-aged and older adults, particularly among those aged 35–69 years, with marked rises across the 35–39, 40–44, 45–49, 50–54, and older age groups, where percentage increases exceeded 500% in several strata. However, these large relative changes should be interpreted in the context of low baseline rates in earlier years, particularly in older age groups. Incidence patterns were similarly heterogeneous, with pronounced increases in middle-aged adults (30–59 years), while younger age groups showed declining or modest trends; notably, incidence decreased among individuals aged 15–19 and 20–24 years. At the same time, substantial spikes in older age groups—especially among those aged ≥65 years—reflect very small baseline values and should be interpreted with caution. Mortality and fatal burden indicators (DALYs and YLLs) declined across most younger and middle-aged groups, consistent with improvements in survival, but showed mixed or slightly increasing trends in older age groups, suggesting a shift in disease burden toward aging populations living with HIV. Non-fatal burden (YLDs) increased across most adult age groups, reinforcing the transition toward chronic disease management. In Central Asia, broadly similar but more moderate patterns were observed. Prevalence and incidence increased across adult age groups, particularly among those aged 30–59 years, although the magnitude of change was lower than in Kazakhstan. As in Kazakhstan, extreme percentage increases in older age groups were driven by low baseline values. In contrast, global patterns differed substantially, with consistent declines in incidence across all age groups and sustained reductions in mortality and DALYs. However, prevalence increased in older populations, particularly among individuals aged ≥45 years. Global and Central Asia values are provided in Supplementary Figure S1.

### 3.4. Projections to 2030 Under Current Trend Assumptions

**Figure 4:**
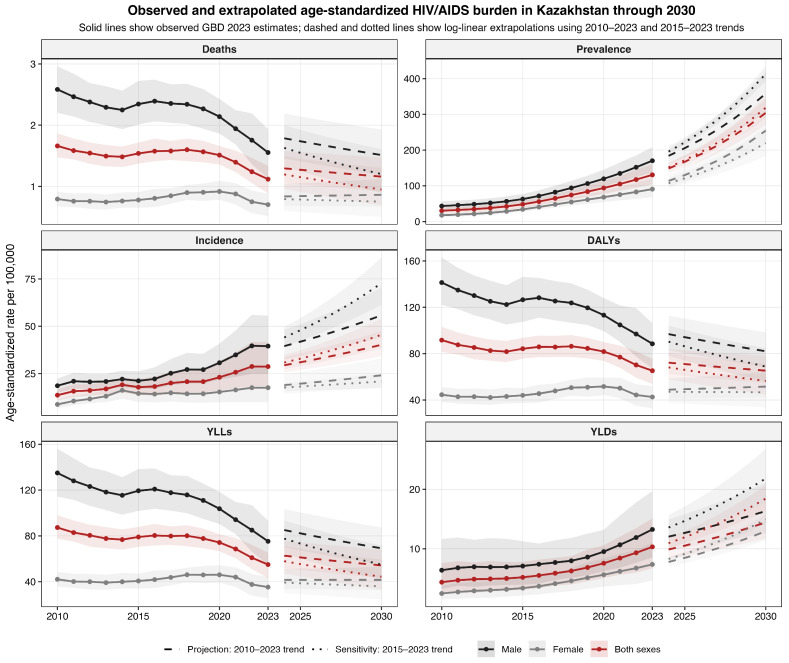
Observed and projected age-standardized HIV/AIDS burden in Kazakhstan, 2010–2030.

Projected trends indicate a continued divergence between non-fatal and fatal components of HIV/AIDS burden in Kazakhstan through 2030 (Figure 4). Age-standardized prevalence is projected to increase substantially across all sex groups. Among males, prevalence is projected to rise from 170.2 in 2023 to 358.1 by 2030, while among females, it is projected to increase from 90.7 to 254.5. For both sexes combined, prevalence is projected to reach 304.4 by 2030, representing a continuation of the upward trajectory observed during the study period. Similarly, incidence is projected to increase further, particularly among males, where rates are projected to rise from 39.5 in 2023 to 55.8 by 2030. Among females, incidence is projected to increase from 17.5 to 24.1, and among both sexes, from 28.7 to 40.2. These projections illustrate potential trajectories under a continuation of observed trends and should not be interpreted as forecasts; they do not account for future changes in testing coverage, PrEP uptake, ART access, migration, or policy. In contrast, mortality and fatal burden indicators are projected to continue declining. Age-standardized death rates among males are projected to decrease from 1.6 in 2023 to 1.5 in 2030, while among females, rates are projected to increase slightly from 0.7 to 0.9, resulting in a relatively stable overall pattern for both sexes (1.1 to 1.2). More pronounced declines are observed in YLLs, with male rates decreasing from 75.2 in 2023 to 69.1 in 2030, and among both sexes from 54.9 to 54.2, indicating continued reductions in premature mortality. DALYs are also projected to decline modestly, with male rates decreasing from 88.5 in 2023 to 82.1 in 2030 and both-sex rates remaining relatively stable (65.3 to 65.3). In contrast, non-fatal burden is projected to increase, with YLDs rising from 13.2 to 16.3 among males, from 7.4 to 13.0 among females, and from 10.3 to 14.4 among both sexes.

## 4. Discussion

This study provides a population-level assessment of HIV/AIDS burden in Kazakhstan from 2010 to 2023, demonstrating a marked increase in age-standardized prevalence and incidence alongside substantial declines in mortality and fatal burden. These patterns indicate a transition toward a chronic and expanding epidemic, with projections to 2030 suggesting continued growth in prevalence and incidence and relatively modest improvements in mortality compared with regional and global trends.

At the regional level, limited access to PrEP and combination prevention services remains a major barrier to epidemic control [15]. Across EECA, prevention programs are often constrained by insufficient public funding, limited technical capacity, and the high cost of service delivery [5]. As a result, the unmet need for PrEP (the PrEP gap) in the region has been estimated at approximately 45%, substantially exceeding levels observed in the European Union (17.4%), reflecting major disparities in prevention coverage [16].

Kazakhstan presents a somewhat different policy environment. The country introduced PrEP in May 2021 and incorporated it into the national list of medicines for free and subsidized outpatient provision, targeting high-risk populations [17]. PrEP access has been gradually expanding. Earlier programmatic reports documented rapid scale-up, with the number of people taking PrEP increasing from approximately 200 in November 2022 to 2000 by June 2023, indicating a ten-fold increase within six months [18]. By July 2024, 7084 people had received PrEP, including 3218 people who initiated PrEP since the beginning of 2024 [19]. By December 2024, the program had achieved approximately 20.1% coverage, with 1936 of 9630 men who have sex with men (MSM) at substantial risk receiving PrEP [20]. Under Kazakhstan’s standard of care, ART, HIV testing, clinical monitoring, and prevention services are provided free of charge and are publicly funded [21]. Despite these advances, the continued increase in incidence and projected growth in burden suggest that current prevention strategies have not yet achieved sufficient population-level impact. This gap likely reflects limitations in program coverage, particularly among key populations, as well as barriers related to stigma, service accessibility, and challenges in building trust and sustaining engagement in care [10,22,23].

The persistence of HIV transmission in Kazakhstan can be better understood through a socio-ecological framework, in which individual risk behaviors are shaped by broader structural and contextual factors [24]. At the individual and interpersonal levels, ongoing transmission is associated with high-risk behaviors, including injection drug use and condomless sex. The increasing use of novel psychoactive substances and the rise of “chemsex” practices—defined as the use of psychoactive substances to enhance sexual activity—have been linked to higher-risk sexual behaviors and increased HIV transmission in multiple settings [9,25,26,27]. These dynamics may play an underrecognized role in sustaining incidence, particularly among urban and marginalized populations. In addition, high rates of depression, anxiety, and other mental health challenges among key populations may further exacerbate these risks [28,29].

At the community level, stigma and discrimination toward key populations and people living with HIV remain significant barriers to accessing prevention and treatment services. At the structural level, legislative and policy environments further shape HIV vulnerability and service engagement. Restrictions on public discussion of LGBTQ-related issues, including the ban on LGBTQ+ “propaganda” [30], may reinforce stigma, reduce the visibility of sexual minority communities, and constrain the delivery of tailored HIV prevention messages [31]. These conditions can limit PrEP uptake, weaken peer-led outreach, and reduce trust in public health services among gay, bisexual, and other men who have sex with men, as well as other sexual and gender minorities [32]. Importantly, recent legislative changes may also affect sex workers, another key population in Kazakhstan’s HIV response. Cordingley et al. highlighted concerns that Law No. 110-VIII on Countering Human Trafficking may blur the distinction between consensual sex work and trafficking, thereby increasing policing, legal uncertainty, and fear among sex workers and service providers [33]. Such legal ambiguity may push sex work into less visible settings, disrupt NGO- and clinic-based outreach, and reduce access to HIV testing, STI treatment, condoms, harm reduction services, and PrEP. Therefore, the observed persistence of HIV incidence in Kazakhstan should be interpreted not only through biomedical prevention coverage, but also through structural barriers that limit the reach and acceptability of prevention services among key populations. Broader geopolitical and socioeconomic factors, including regional instability, migration, and economic pressures, may further increase vulnerability and disrupt continuity of prevention and care.

The findings of this study suggest that Kazakhstan’s HIV response should move beyond nominal service availability toward differentiated, low-threshold, and key population-centered delivery models. Although PrEP is now available across cities in Kazakhstan, uptake may remain limited if services are perceived as stigmatizing, inconvenient, insufficiently confidential, or disconnected from the communities at greatest risk.

The projected continued increase in incidence under current trend assumptions, alongside the documented PrEP gap in EECA [17], suggests that existing prevention efforts have not yet achieved sufficient population-level impact. To address this, PrEP scale-up should prioritize peer-led and community-based navigation, PrEP counseling in trusted non-specialist settings, integration with HIV self-testing and STI services, digital appointment systems, discreet refill pathways, and targeted demand-generation campaigns for gay, bisexual, and other men who have sex with men, sex workers, people who inject drugs, migrants, and younger adults. Long-acting PrEP modalities [34,35,36], when approved, affordable, and programmatically feasible, may also help address barriers related to pill burden, privacy, and adherence. However, long-acting PrEP options should be introduced alongside community education and provider training to avoid widening existing access gaps [37]. Harm reduction should similarly be strengthened through integrated service packages that combine syringe service programs, opioid agonist therapy referral, HIV/HCV/STI testing, condom distribution, PrEP referral, and direct linkage to ART in the same low-threshold settings. Evidence from Kazakhstan indicates that stigma can also occur within harm reduction and healthcare environments, including denial of services, perceived negative attitudes, unnecessary precautions, and breaches of confidentiality; therefore, expanding services without addressing provider behavior may be insufficient [10,23,31,38]. Strengthening the care cascade should include routine opt-out HIV testing in primary care, STI, tuberculosis, antenatal, and harm reduction settings; same-day linkage to ART; active follow-up after diagnosis; and peer or case-manager support for individuals at risk of disengagement. These measures are particularly relevant because healthcare-provider stigma remains substantial in Kazakhstan: a recent mixed-methods study found high levels of stigmatizing attitudes toward people living with HIV and key populations, with many providers reporting unwillingness to provide care to sex workers, people who inject drugs, and MSM [10]. Stigma-reduction efforts should therefore move beyond one-time informational training and use interactive, skills-based modules addressing HIV transmission, U=U, confidentiality, medical ethics, and respectful care for key populations. Targeted outreach should also use locally adapted digital and youth-led strategies. A recent Kazakhstan study showed that adolescents and young adults were willing to produce creative crowdsourced HIV stigma-reduction and testing messages, suggesting that peer-generated digital content may be a feasible way to improve testing demand and normalize prevention among younger populations [39]. Taken together, these findings indicate that epidemic control in Kazakhstan will require not only expanded biomedical prevention but also community trust-building, differentiated service delivery, provider stigma reduction, and legal and policy environments that allow key populations to access prevention and care without fear of disclosure, discrimination, or punitive consequences.

Best practices from other settings suggest that integrated approaches combining biomedical, behavioral, psychological, and structural interventions are most effective in achieving the UNAIDS 95–95–95 targets [40]. These include community-based testing, peer-led interventions, digital outreach, and policy reforms that promote inclusivity and reduce barriers to care.

This study has several strengths, including the use of standardized GBD 2023 estimates, enabling consistent comparisons across countries and over time. The inclusion of multiple burden indicators, along with sex-specific and age-specific analyses, provides a comprehensive understanding of HIV epidemiology in Kazakhstan. The addition of projection analyses further enhances the study by offering insight into potential future trends.

Several limitations should be considered when interpreting these findings. The GBD estimates are modeled outputs that integrate surveillance, registry, vital registration, and covariate data and may be sensitive to assumptions regarding incidence, mortality, disease progression, and cause-of-death redistribution, particularly in EECA settings where late diagnosis, underdiagnosis, and incomplete surveillance remain important concerns. Although uncertainty intervals are reported, they mainly reflect model-based statistical uncertainty and may not fully capture real-world biases related to underreporting among stigmatized and hard-to-reach populations, including MSM, people who inject drugs, sex workers, migrants, and transgender individuals. The analysis was also limited by the structure of the GBD data, which does not allow disaggregation by key population, risk behavior, gender-diverse identity, or subnational region; therefore, important heterogeneity in HIV burden within Kazakhstan may be obscured. The projections should be interpreted cautiously because they rely on log-linear extrapolation and do not account for future changes in prevention coverage, treatment access, funding, migration, policy environments, or behavioral patterns. Regional comparisons should also be interpreted with caution because the GBD Central Asia grouping includes countries that are not traditionally considered part of Central Asia, introducing heterogeneity in epidemiological and health system contexts. Finally, the ecological design precludes causal inference and does not allow assessment of individual-level determinants of HIV acquisition, diagnosis, treatment engagement, or mortality. Taken together, these limitations highlight priority areas for future research, including prospective cohort studies with disaggregated data by key population, behavioral surveys linked to surveillance systems, and comparative analyses incorporating health system and prevention intervention data to enable more robust causal inference.

## 5. Conclusions

In conclusion, the HIV/AIDS burden in Kazakhstan is characterized by a dual epidemiological pattern of declining mortality and increasing incidence and prevalence, reflecting a transition toward a chronic and expanding epidemic. Under current trend assumptions, projections indicate continued divergence between non-fatal and fatal burden components through 2030. However, these trajectories are conditional on the continuation of observed trends and do not reflect the potential impact of future policy or programmatic changes. Compared with regional and global trends, Kazakhstan shows slower progress in reducing diagnosed incidence, with the burden increasingly concentrated among adult populations and key groups. Despite improvements in treatment access and the introduction of PrEP, persistent structural, social, and behavioral factors are plausible contributors to sustained transmission; however, because this study is ecological and based on modeled aggregate data, direct causal attribution is not possible. Strengthening prevention strategies, expanding coverage of evidence-based interventions, and addressing structural barriers will be essential for achieving epidemic control and meeting the 95–95–95 targets.

## Figures and Tables

**Table 1:** Percent change in age-standardized HIV/AIDS rates in Kazakhstan, Central Asia, and globally, 2010–2023.

Measure	Kazakhstan			Central Asia			Global		
	2010 Rate (95% UI)	2023 Rate (95% UI)	% Change, 2010–2023	2010 Rate (95% UI)	2023 Rate (95% UI)	% Change, 2010–2023	2010 Rate (95% UI)	2023 Rate (95% UI)	% Change, 2010–2023
Male									
Deaths	2.6 (2.2–3.0)	1.6 (1.1–1.9)	−39.9%	2.2 (2.0–2.4)	1.5 (1.2–1.7)	−33.7%	16.9 (15.8–18.6)	10.0 (8.7–11.5)	−40.8%
Prevalence	43.4 (32.2–56.3)	170.2 (136.8–208.3)	292.6%	40.4 (30.5–53.7)	99.3 (83.3–120.1)	145.7%	377.8 (355.7–399.9)	445.1 (410.8–478.5)	17.8%
Incidence	18.6 (14.8–22.4)	39.5 (24.8–55.6)	112.5%	9.8 (7.5–12.7)	22.5 (13.7–34.7)	129.2%	32.6 (31.1–34.1)	25.5 (23.3–28.5)	−21.7%
DALYs	141.4 (122.2–163.0)	88.5 (67.2–106.4)	−37.4%	120.9 (111.1–131.8)	81.1 (67.6–94.0)	−32.9%	935.3 (872.3–1025.9)	540.0 (477.4–606.4)	−42.3%
YLLs	135.0 (114.2–156.1)	75.2 (54.7–93.2)	−44.2%	114.9 (105.2–125.9)	73.0 (60.8–86.3)	−36.5%	884.4 (824.3–969.6)	494.1 (435.2–562.1)	−44.1%
YLDs	6.4 (3.3–11.6)	13.3 (8.6–19.7)	107.1%	6.0 (3.3–10.1)	8.1 (5.4–11.6)	35.7%	51.0 (36.3–65.7)	45.9 (33.4–60.7)	−9.9%
Female									
Deaths	0.8 (0.7–0.9)	0.7 (0.5–0.9)	−11.4%	0.8 (0.8–0.9)	0.9 (0.7–1.0)	2.2%	16.9 (15.5–18.5)	9.8 (8.3–11.4)	−42.1%
Prevalence	17.7 (12.7–23.3)	90.7 (67.9–121.5)	412.8%	20.7 (15.2–27.5)	72.4 (59.3–88.1)	249.9%	452.9 (437.4–467.0)	556.7 (528.5–587.5)	22.9%
Incidence	8.7 (6.8–11.2)	17.6 (9.9–28.7)	101.1%	5.9 (3.9–7.9)	14.7 (9.8–21.3)	149.9%	36.8 (35.4–38.4)	26.7 (24.1–30.3)	−27.5%
DALYs	44.6 (38.2–51.2)	42.6 (33.0–51.8)	−4.4%	47.2 (43.1–51.7)	50.2 (42.0–58.6)	6.5%	986.7 (911.7–1074.3)	558.3 (488.6–640.5)	−43.4%
YLLs	42.1 (35.6–48.5)	35.3 (25.7–44.1)	−16.2%	44.2 (39.8–48.6)	44.0 (36.2–52.9)	−0.6%	923.8 (848.1–1014.9)	502.1 (433.2–583.8)	−45.7%
YLDs	2.5 (1.5–3.8)	7.4 (4.7–11.1)	196.8%	2.9 (1.7–4.5)	6.2 (4.2–8.7)	112.5%	62.8 (46.4–80.9)	56.3 (40.5–75.3)	−10.5%
Both sexes									
Deaths	1.7 (1.5–1.9)	1.1 (0.9–1.3)	−32.7%	1.5 (1.4–1.6)	1.2 (1.0–1.3)	−23.5%	16.9 (15.8–18.3)	9.9 (8.6–11.3)	−41.5%
Prevalence	30.2 (22.3–39.4)	130.6 (103.8–160.9)	332.1%	30.4 (22.9–40.3)	85.9 (71.4–103.4)	182.9%	414.6 (401.5–429.6)	499.7 (474.8–524.3)	20.5%
Incidence	13.6 (10.9–16.6)	28.7 (18.3–41.7)	111.0%	7.8 (5.7–10.2)	18.6 (12.0–27.8)	138.6%	34.6 (33.4–35.7)	26.1 (23.9–28.9)	−24.7%
DALYs	91.7 (81.5–103.1)	65.3 (53.9–75.5)	−28.8%	83.3 (77.3–89.3)	65.5 (57.1–72.5)	−21.4%	960.6 (897.6–1038.8)	548.7 (485.1–618.4)	−42.9%
YLLs	87.3 (77.5–98.1)	55.0 (43.2–65.2)	−37.1%	78.9 (73.4–84.1)	58.3 (51.1–65.3)	−26.0%	903.8 (843.1–978.0)	497.7 (439.1–568.7)	−44.9%
YLDs	4.4 (2.4–7.6)	10.3 (6.7–15.1)	135.5%	4.4 (2.5–7.2)	7.2 (4.7–10.1)	62.4%	56.8 (41.6–72.3)	51.0 (37.1–67.6)	−10.3%

Abbreviations: UI, uncertainty interval; DALYs, disability-adjusted life years; YLLs, years of life lost; YLDs, years lived with disability. All rates are age-standardized per 100,000 population. Percent change was calculated as [(2023 rate − 2010 rate)/2010 rate] × 100. Decimal places are consistent to one decimal place throughout.

**Table 2:** Annual percentage change in age-standardized HIV/AIDS rates in Kazakhstan, Central Asia, and globally, 2010–2023.

Measure	Kazakhstan		Central Asia		Global	
	APC % (95% CI)	*p*-Value	APC % (95% CI)	*p*-Value	APC % (95% CI)	*p*-Value
Male						
Deaths	−2.7 (−3.9 to −1.6)	<0.001	−2.6 (−3.4 to −1.9)	<0.001	−3.9 (−4.1 to −3.8)	<0.001
Prevalence	11.8 (10.9 to 12.6)	<0.001	7.5 (7.0 to 8.0)	<0.001	1.3 (1.3 to 1.4)	<0.001
Incidence	5.9 (4.7 to 7.1)	<0.001	6.7 (6.3 to 7.2)	<0.001	−1.9 (−2.0 to −1.8)	<0.001
DALYs	−2.7 (−3.6 to −1.9)	<0.001	−2.7 (−3.2 to −2.1)	<0.001	−4.1 (−4.3 to −3.9)	<0.001
YLLs	−3.4 (−4.5 to −2.3)	<0.001	−3.0 (−3.7 to −2.3)	<0.001	−4.4 (−4.5 to −4.2)	<0.001
YLDs	5.2 (3.9 to 6.5)	<0.001	1.8 (0.8 to 2.9)	0.003	−0.8 (−0.9 to −0.7)	<0.001
Female						
Deaths	0.5 (−0.7 to 1.8)	0.371	1.1 (−0.1 to 2.2)	0.067	−3.9 (−4.5 to −3.3)	<0.001
Prevalence	14.4 (13.5 to 15.3)	<0.001	10.7 (10.1 to 11.2)	<0.001	1.7 (1.5 to 1.8)	<0.001
Incidence	4.1 (2.5 to 5.7)	<0.001	5.9 (3.7 to 8.2)	<0.001	−2.5 (−2.6 to −2.4)	<0.001
DALYs	0.9 (−0.1 to 1.9)	0.082	1.3 (0.3 to 2.3)	0.09	−4.1 (−4.7 to −3.5)	<0.001
YLLs	0.0 (−1.2 to 1.2)	0.968	0.8 (−0.3 to 2.0)	0.145	−4.4 (−5.00 to −3.8)	<0.001
YLDs	8.9 (8.2 to 9.5)	<0.001	6.0 (5.6 to 6.4)	<0.001	−0.8 (−0.9 to −0.7)	<0.001
Both sexes						
Deaths	−1.8 (−2.9 to −0.7)	0.005	−1.4 (−2.3 to −0.5)	0.004	−3.9 (−4.3 to −3.6)	<0.001
Prevalence	12.7 (12.1 to 13.4)	<0.001	8.8 (8.4 to 9.1)	<0.001	1.5 (1.4 to 1.6)	<0.001
Incidence	5.4 (4.5 to 6.2)	<0.001	6.4 (5.3 to 7.6)	<0.001	−2.2 (−2.3 to −2.1)	<0.001
DALYs	−1.7 (−2.5 to −0.8)	0.001	−1.3 (−2.00 to −0.6)	0.001	−4.1 (−4.5 to −3.8)	<0.001
YLLs	−2.4 (−3.5 to −1.3)	<0.001	−1.7 (−2.5 to −0.9)	<0.001	−4.4 (−4.8 to −4.00)	<0.001
YLDs	6.5 (5.4 to 7.6)	<0.001	3.5 (2.7 to 4.3)	<0.001	−0.8 (−0.9 to −0.7)	<0.001

Abbreviations: APC, annual percent change; CI, confidence interval; DALYs, disability-adjusted life years; YLLs, years of life lost; YLDs, years lived with disability. APC was derived from log-linear regression of the natural log of age-standardized rate on calendar year; *p*-values were based on two-sided *t*-tests.

## Data Availability

Publicly available datasets were analyzed in this study. These data can be found here https://vizhub.healthdata.org/gbd-results/ (accessed on 28 January 2026).

## Supplementary Materials

The following supporting information can be downloaded at https://www.mdpi.com/article/10.3390/tropicalmed11070171/s1, Supplementary Figure S1. Percent change in age-specific HIV/AIDS rates, Kazakhstan, Central Asia, and globally, 2010–2023 (both sexes).
