# Supplementary figures and images for: Trends and Projected Burden of HIV/AIDS in Kazakhstan, 2010–2030: A Comparative Analysis Using GBD 2023 Estimates
Source: Trop Med Infect Dis. 2026 Jun 24;11(7):171. doi: 10.3390/tropicalmed11070171 (PMC13417225; doi:10.3390/tropicalmed11070171)

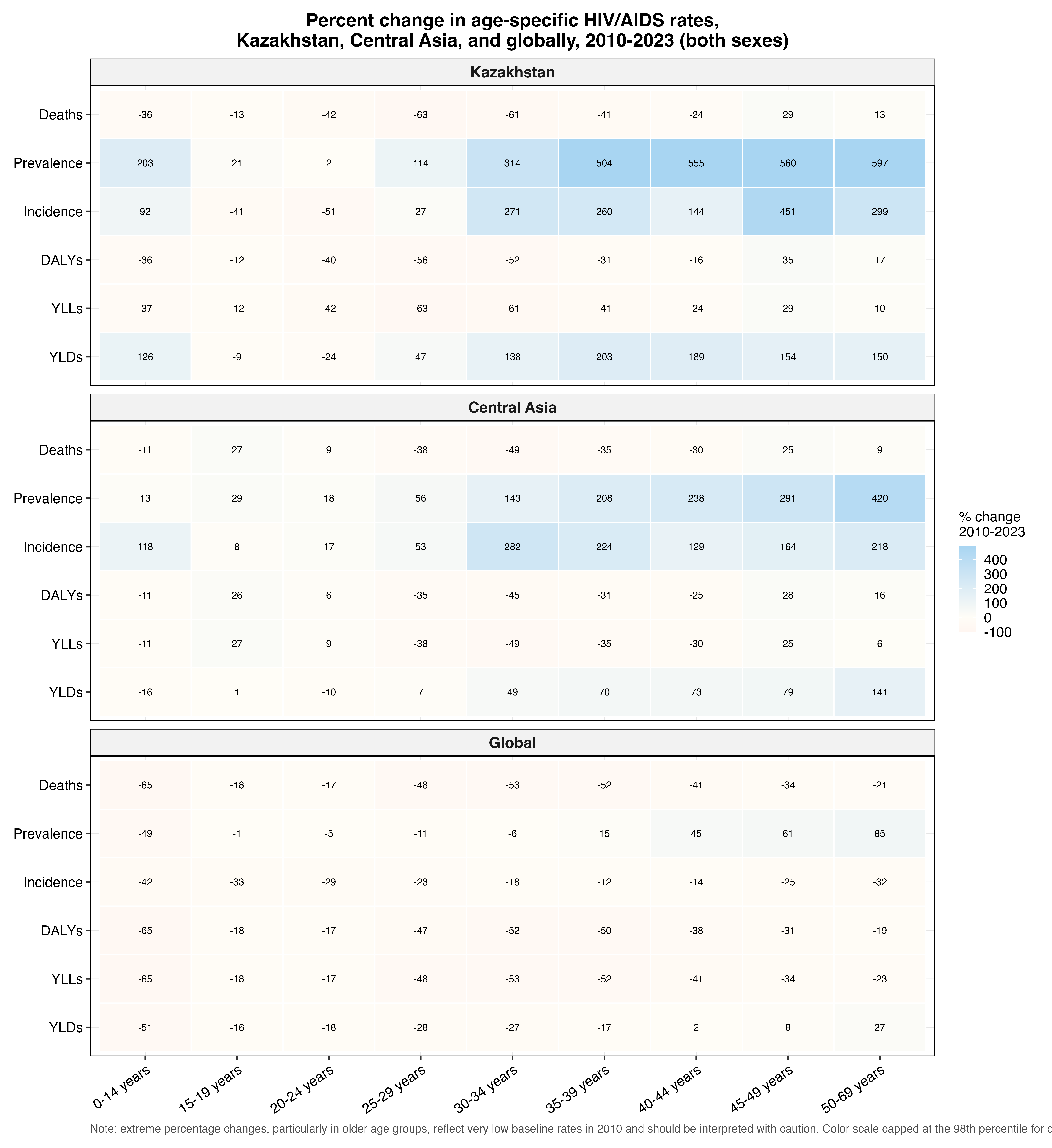

Supplement: Supplementary file 1 [file tropicalmed-11-00171-s001.zip › tropicalmed-4377871-supplementary.tiff]
